# Commensal Microbiota Contributes to Chronic Endocarditis in *TAX1BP1* Deficient Mice

**DOI:** 10.1371/journal.pone.0073205

**Published:** 2013-09-27

**Authors:** Satoko Nakano, Emi Ikebe, Yoshiyuki Tsukamoto, Yan Wang, Takashi Matsumoto, Takahiro Mitsui, Takaaki Yahiro, Kunimitsu Inoue, Hiroaki Kawazato, Aiko Yasuda, Kanako Ito, Shigeo Yokoyama, Naohiko Takahashi, Mitsuo Hori, Tatsuo Shimada, Masatsugu Moriyama, Toshiaki Kubota, Katsushige Ono, Wataru Fujibuchi, Kuan-Teh Jeang, Hidekatsu Iha, Akira Nishizono

**Affiliations:** 1 Department of Microbiology, Faculty of Medicine, Oita University, Yufu, Oita, Japan; 2 Department of Ophthalmology, Faculty of Medicine, Oita University, Yufu, Oita, Japan; 3 Department of Molecular Pathology, Faculty of Medicine, Oita University, Yufu, Oita, Japan; 4 Department of Pathophysiology, Faculty of Medicine, Oita University, Yufu, Oita, Japan; 5 Research Promotion Institute, Faculty of Medicine, Oita University, Yufu, Oita, Japan; 6 Department of Internal Medicine II, Faculty of Medicine, Oita University, Yufu, Oita, Japan; 7 Department of Diagnostic Pathology, Faculty of Medicine, Oita University, Yufu, Oita, Japan; 8 Department of Laboratory Examination and Diagnostics, Faculty of Medicine, Oita University, Yufu, Oita, Japan; 9 Division of Hematology, Ibaragi Prefectural Central Hospital, Kasama, Ibaragi, Japan; 10 Department of Health Science, Oita University School of Nursing, Yufu, Oita, Japan; 11 Department of Cell Growth and Differentiation, Center for iPS Cell Research and Application, Kyoto University, Kyoto, Japan; 12 Laboratory of Molecular Microbiology, National Institute of Allergy and Infectious Diseases, National Institutes of Health, Bethesda, Maryland, United States of America; University of Hong Kong, Hong Kong

## Abstract

Tax1-binding protein 1 (Tax1bp1) negatively regulates NF-κB by editing the ubiquitylation of target molecules by its catalytic partner A20. Genetically engineered *TAX1BP1*-deficient (KO) mice develop age-dependent inflammatory constitutions in multiple organs manifested as valvulitis or dermatitis and succumb to premature death. Laser capture dissection and gene expression microarray analysis on the mitral valves of *TAX1BP1-*KO mice (8 and 16 week old) revealed 588 gene transcription alterations from the wild type. *SAA3* (serum amyloid A3), *CHI3L1*, *HP*, *IL1B* and *SPP1/OPN* were induced 1,180-, 361-, 187-, 122- and 101-fold respectively. *WIF1* (Wnt inhibitory factor 1) exhibited 11-fold reduction. Intense Saa3 staining and significant I-κBα reduction were reconfirmed and massive infiltration of inflammatory lymphocytes and edema formation were seen in the area. Antibiotics-induced ‘germ free’ status or the additional *MyD88* deficiency significantly ameliorated *TAX1BP1-*KO mice's inflammatory lesions. These pathological conditions, as we named ‘pseudo-infective endocarditis’ were boosted by the commensal microbiota who are usually harmless by their nature. This experimental outcome raises a novel mechanistic linkage between endothelial inflammation caused by the ubiquitin remodeling immune regulators and fatal cardiac dysfunction.

## Introduction

The transcription factor NF-κB is essential for the regulation of the innate and adaptive immune responses. NF-κB is activated in response to a wide variety of stimuli, such as inflammation, DNA damage, or nociception [1], [2], and is involved in embryogenesis and multiple tissue development [3]. The NF-κB family comprises five proteins including RelA (p65), RelB, c-Rel, NF-κB1, and NF-κB2, and their transcriptional activities are tightly controlled to ensure their transient signaling in response to specific stimuli. The NF-κB signaling cascade is usually triggered by sensor molecules, such as toll-like receptor (TLR) family proteins. These proteins can identify the presence of a wide range of microorganisms and then transmit that information through phosphorylation relays to downstream kinases, which eventually culminate at the I-κB kinase (IKK). IKK activates NF-κB via phosphorylation of inhibitory I-κB proteins (primarily I-κBα), which leads to its ubiquitylation and degradation by the 26S proteasome complex and allows NF-κB to enter the nucleus. I-κB is induced by NF-κB to function in a negative feedback loop that terminates NF-κB signaling. Aberrant activation of NF-κB has been linked to several pathological features such as allergic responses, autoimmune diseases, septic shock, and carcinogenesis in a variety of organs [4].

In addition to I-κB, deubiquitinase A20 (also referred to as TNFα-induced protein 3 or TNFAIP3) targets important signaling intermediates upstream of I-κB to terminate NF-κB activation [5], [6]. A20 cleaves Lys63 (K63)-linked polyubiquitin chains on overlapping substrates, such as E3 ubiquitin ligase TRAF6 and adaptor molecule RIP1, with the help of the substrate-specific adaptor Tax1-binding protein 1 (Tax1bp1 [7], [8]). Tax1bp1 intrinsically regulates NF-κB by recruiting A20 to the target molecules to remove their polyubiquitin chains, which play important roles in their assembly into the IKK complex [8], [9]. Deficiencies in A20 or Tax1bp1 lead to uncontrolled and spontaneous systemic inflammation in mice as a result of unchecked NF-κB signaling [8], [10].

Tax1bp1 was originally identified as a host cell factor that binds to the encoded protein of human T-lymphotropic virus type 1 (HTLV-1), known as Tax1 [7]. Tax1 is a potent activator of NF-κB and a major pathogenic factor in HTLV-1 associated diseases (HAD), such as HTLV-1 associated myelopathy (HAM) or HTLV-1 uveitis (HU [11]), and adult T-cell leukemia (ATL [12]). Tax1 interrupts the ability of Tax1bp1 to connect to and recruit A20 to target molecules and thus evokes persistent NF-κB activation [13], [14]. Tax1 also activates NF-κB by binding to the NF-κB essential modulator (NEMO), a regulatory subunit of IKK [15]. The aberrant activation of NF-κB in HADs can therefore be attributed to Tax1, which leads to Tax1bp1 dysfunction, over-activation of IKK, or both. Epidemiological studies provide support for a close link between HTLV-1 infection and HAD or other inflammatory diseases such as Sjögren's syndrome [16], vascular dementia [17], and atherosclerosis [18]. Moreover, recent accumulating evidence strongly suggests that several mutations in the *A20* locus are primarily responsible for the development of Crohn's disease, rheumatoid arthritis, systemic lupus erythematosus, psoriasis and type 1 diabetes [19].

For research purposes, we established *TAX1BP1*-deficient (-KO) mice, which display exacerbation of inflammation (characterized as valvulitis and dermatitis) in an age-dependent manner in addition to functional inadequacies manifested in growth retardation and premature death [8]. To elucidate the molecular mechanisms underlying the manifestation of inflammatory symptoms and their link to premature or possible cardiac abnormalities induced by *TAX1BP1*-deficiency, we performed a series of pathological evaluations using *TAX1BP1*-KO mice: (1) laser capture microdissection (LCM)- and gene expression microarray-based profiling of the mitral valves, which was reevaluated using real-time polymerase chain reaction (RT-PCR); (2) multiplex cytokine and chemokine quantitation in sera on systemic inflammatory constitution; (3) histochemical and electron microscopic analyses of multiple pathogenic foci; and (4) antibiotic treatments and cross experimentation with *MyD88*-deficient mice [20] to examine the role of commensal microbiota in the pathogenesis of *TAX1BP1*-KO mice.

From our experimental data, we conclude that systemic inflammation and cardiac structural abnormalities in *TAX1BP1*-KO mice originated from commensal microbiota, which are usually harmless in nature. Furthermore, these results indicate a potential risk to asymptomatic HTLV-1 carriers, which should be addressed by further clinical research.

## Materials and Methods

### Animals

*TAX1BP1-*KO mice having replaced their exon 17 region with CMV-driven NEO gene in reverse orientation [8] and their wild-type (WT) littermates as controls were analyzed throughout the experiment. These strains are maintained as F9 or advanced generations of C57BL/6CrSlc or the original 129/+ Ter/SvJcl. *MyD88* deficient mice are kind gifts from professor Hitoshi Nakashima from Fukuoka University [21]. Homozygous *TAX1BP1*-KO mice were crossbred with homozygous *MyD88*-KO background to generate *MyD88/TAX1BP1*-KO mutants. Each of the targeted loci was evaluated by PCR. These mice were bred and maintained under specific pathogen-free (SPF) conditions at the animal facility of Oita University Faculty of Medicine. All the mice related manipulations were performed with protocols approved by the animal ethics committee at the Oita University (Justified numbers, daily care, treatment and euthanasia procedures).

### Laser capture microdissection

Three mitral valves from 8 or 16 week old (-wk) male*TAX1BP1-*KO and their WT littermates were collected by Arcturus XT laser capture microdissection system according to a manufacture's directions.

### RNA Isolation and gene expression microarray analysis

Total RNAs were purified from the mitral valves using RNeasy mini kit (Qiagen). RNA quantity and purity were evaluated using a NanoDrop 2000 (NanoDrop Technologies). All RNA samples were labeled, linearly amplified by Low Input Quick Amp Labeling Kit and RNA Spike-In Kit then analyzed with Whole Mouse Genome Microarray Kit (Agilent). Signal intensities were quantitated with laser confocal scanner and analyzed with Feature Extraction software (Version 10.7.3.1, Agilent) and R statistical package (Version 2.15.1). Probe set data were median-normalized per chip. Empirical Bayesian method controlling for false discovery rate (FDR: <3% and logFC >1.0 [22]) for comparison of differentially expressed between *TAX1BP1-*KO mice and their WT. Principal Component Analysis (PCA) for the systematic trend examination, heatmaps by R Software and volcano plot analysis were applied to identify the single mRNA differentially expressed in *TAX1BP1-*KO mice (log2-fold expression change on the x-axis and t test p values on the y-axis, negative log). Each dot represents a single probe. The complete gene expression dataset can be viewed in the Gene Expression Omnibus (GEO) repository accession number GSE43932 (www.ncbi.nlm.nih.gov/geo/query/acc.cgi?acc=GSE43932).

### Quantitative real time-polymerase chain reaction (RT-PCR)

Taqman quantitative RT-PCR was performed to validate a subset of genes. Random hexamer-primed cDNA templates were synthesized from purified (RNAs ReverTra Ace®, TOYOBO). The output of RT-PCR reactions were quantitated with LightCycler® R 480 System (Roche). Primer sequences were listed in Table 1. Each reaction was run in triplicate with endogenous control *GAPDH* on the same reaction plate.

**Table 1:** Primer sequences.

*SAA3*	acagcctctctggcatcg	atgctcggggaactatgat	#26
*TAX1BP1*	ataaaaatgtgtaatagtcacgagcag	cactccaaagattgggttgg	#56
*EFCAB2*	tgtccgtcgtggctatgac	cctgcttcaccaccttcttg	#80
*GAPDH*	tcgaccatgaatcgaataataca	tgcagctctccttcagtcg	#89

### Multiple cytokine & chemokine quantitation

The 3-, 8-, 16- and 32-wk male *TAX1BP1-*KO and their WT littermates were anesthetized and an aliquot of serum (12.5 μl) from heart blood were collected (n = 5/groups). Quantitation of 23 cytokines and chemokines was performed by a multiplex ELISA system (Bio-Plex, BioRad) and analyzed by the Bio-Plex Manager Software 6.1 (Bio-Rad) with a five-parameter curve-fitting algorithm for standard curve calculations.

### Immunohistochemistry

A standard avidin-biotin-peroxidase technique or hematoxylin and eosin (HE) staining were employed for Saa3 and I-κBα staining or morphological observation of heart, liver and skin tissues of 8- or 16-wk male *TAX1BP1-*KO and their WT littermates (n = 5/groups). Rabbit polyclonal anti-Saa3 antibody (ab59736, abcum), rabbit monoclonal anti-I-κBα antibody (ab32518, abcum) or control antibody for visualization of antigens with EnVision + System-HRP Labelled Polymer Anti-Rabbit (Dako). DAB + Liquid (Dako) for positive staining and Mayer's hematoxylin solution for counterstaing. Images were captured with BZ-9000 (KEYENCE). Mice whole eye sections were examined with anti-T6BP antibody (ab22049, abcam). Anti-IgG (H+L), rabbit, goat-poly, DyLight 649 (KPL) was used as secondary antibodies.

### Electron microscopy

For transmission electron microscopy (TEM), mitral valve, atrioventricular node, sinoatrial node and papillary muscles of the left ventricle of 8-, 16-, 60-wk male *TAX1BP1-*KO and their WT littermates (n = 3/groups) were fixed with 2.5% glutaraldehyde/2% paraformaldehyde in a 0.1 M cacodylate buffer (pH7.4) for 3 hr or longer at 4°C. After a washing in the cacodylate buffer, specimens were postfixed in 2% osmium tetroxide in cacodylate buffer for 2 hr, washed with cacodylate buffer, dehydrated with ethanol and embedded in epoxy resin. Thin section specimens (80–90 nm) were then stained with uranyl acetate and lead cystate and examined with TEM H-7650 (at 80 kV, HITACHI).

### Western blotting

Tissues from liver, heart, spleen, muscle, lung, skin, stomach and brain from WT BL6 were lysed with Co-IP buffer [23] and equal amounts of protein solutions (20 μg/lane) were separated by SDS-PAGE and transferred to immobilion membranes (Millipore) and incubated with primary antibodies, T6BP Antibody (sc-15274, Santa Cruz) or anti-Tubulin antibody (ab6160, abcum) and secondary antibodies, donkey anti-goat IgG-HRP (sc-2033, Santa Cruz) or ZyMAX™ Goat anti-Rat IgG(H+L) HRP conjugate (81–9520, invitrogen) and visualized with ECL Western Blotting Detection System (GE Healthcare Lifesciences) and high-performance chemiluminescence film.

### Evaluation of physiological responses to LPS-stimulation

200 μg of *Salmonella typhimurium* lipopolysaccharide (LPS, Sigma) in 100 μl sterile pyrogen-free saline were injected into the footpads of *TAX1BP1-*KO or WT littermates (n = 4/groups). Tissue lysates were prepared from eyeball and the expression of Tax1bp1, I-κBα (anti-I-κBα rabbit mAb, #4812, Cell Signaling Technology) and Tubulin were evaluated by western blotting. Total RNAs were prepared from eyeballs of *TAX1BP1-*KO or WT littermates (n = 4/groups). Taqman quantitative RT-PCR was performed as described above (See Table 2).

**Table 2:** 

*IL6*	gctaccaaactgga tataatcagga	ccaggtagctatgg tactccagaa	#6
*CXCL1*	agactccagccacacactccaa	tgacagcgcagctcattg	#83
*GAPDH*	tcgaccatgaatcgaataataca	tgcagctctccttcagtcg	#89

Sera from peripheral blood samples were collected 0, 6 and 12 hr after LPS injection and quantitated with Bio-Plex Pro™ Mouse Cytokine 23-plex kit.

### Enzyme-linked immunosorbant assay (ELISA)

The amounts of Saa3 and Cxcl13 Sera from 16-wk mice (n = 5/group) were measured with MOUSE SAA-3 ELISA KIT (Millipore) and Mouse CXCL13/BLC/BCA-1 Quantikine ELISA Kit (R&D Systems).

### Telemetric electrocardiogram (ECG)

Sixteen week old male *TAX1BP1*-KO or WT littermates with or without antibiotic treatment (n = 5/group) were monitored with telemetric electrocardiogram. Telemetric transmitter was implanted into the back of mice under aseptic conditions and the muscle layers and the skin were closed with resorbable sutures. Data were acquired at least 72 hour after the implantation with a receiver placed under the cage and a full-disclosure 72 hour recordings were analyzed off-line and the P-Q intervals were evaluated.

### Antibiotic treatment

*TAX1BP1*-KO or WT littermate male mice were first raised with the normal diets and water for 4 weeks, and then, antibiotic group (n = 5/groups) received ampicillin (1 g/L; Wako), vancomycin hydrochloride (500 mg/L; Wako), neomycin trisulfate salt hydrate (1 g/L; Sigma-Aldrich), and metronidazole (1 g/L; Wako) in drinking water for 12 weeks [24]. The non-antibiotic controls were equally raised and maintained except for antibiotics treatment. Both groups of mice were maintained in flexible film isolators under a strict 12-hour light cycle and fed an autoclaved chow diet and tap water ad libitum. Germ free status was verified regularly by ensuring negative cultures from mouse feces in three media types: nutrient agar (Nissui), pourmedia sheep blood agar M70 (Eiken), and Sabouraud agar (Nissui). Microbial colonies were counted after incubation at 37°C for 48 hour (aerobes) or 72 hour (anaerobes). Both groups of mice were anesthetized and sacrificed at the end of 16 weeks experimental period. Daily fluid consumption, body weight, liver function (ALT, AST), renal function (BUN), nutritional status (TG, GLU, TP) and spleen weight (After 10% formalin fixation) were examined. Caecum surface area was measured with Image J (NIH). In general, there were no particular adverse effects on mice through antibiotic treatment.

### Statistical analysis

All numerical data are expressed as means ± SD. Statistical significance was assessed by Student's two-tailed t-test. In the case of ELISA, Statistical analyses were performed by one-way analysis of variance and Steel-Dwass test. Data were considered significant when P<0.05.

## Results

### LCM- and gene expression microarray array-based profiling of the mitral valves in *TAX1BP1*-KO mice and reevaluation by RT-PCR and immunostaining

We have previously observed that the mRNA expression level for several inflammatory cytokines, including IL-1β and TNFα, increases in the cardiac and skin tissues of *TAX1BP1-*KO mice; more importantly, these mice showed mitral valvulitis and premature death compared to their wild-type (WT) littermates. However, the underlying mechanisms involved in these processes remain unknown [8].

To date, information on variations in the levels of gene expression in regions of the heart (more specifically, the mitral valves) showing inflammation in *TAX1BP1*-KO mice is still lacking. This pathologic event is thought to be linked to premature death, which might be brought on by cardiac failure. In the current study, we employed LCM- and gene expression microarray-based techniques to obtain detailed information on the levels of gene expression in organs showing pathological changes. Total RNA was extracted from three independent tissue samples obtained from the mitral valves of 8- or 16- week-old (-wk) male (WT and TAX1BP1-KO) mice by using LCM, which was followed by total RNA extraction. Then, global mRNA expression profiles were analyzed by an Agilent gene expression microarray.

**Figure 1:**
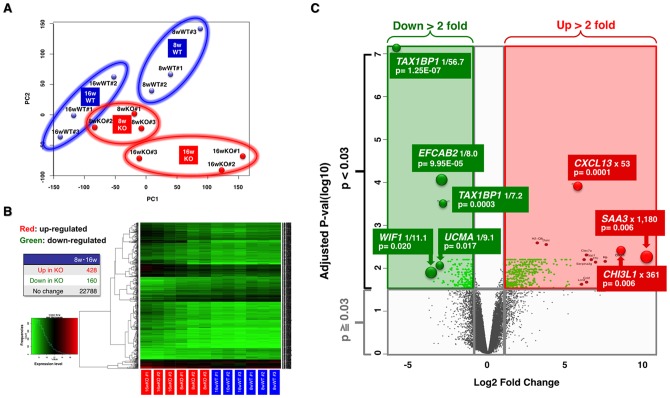
Elevated inflammatory profiles in the multiple organs of *TAX1BP1*-KO mice. Mitral valve tissues from either 8 or 16(-wk) *TAX1BP1*-KO mice or their wild-type littermates were collected by Arcturus XT LCM system and total RNAs were prepared by RNeasy mini kit (Qiagen). Each cDNA pool was generated from the individual RNA sample and gene expression profiles were evaluated using Whole Mouse Genome Microarray Kit (Agilent). **A**) Principal component analysis (PCA) by conditions was performed on R statistical package (Version 2.15.1) and represented as a scatterplots of whole gene expression profiles of 8- or 16-wk *TAX1BP1*-KO mice (8wKO #1- #3 or 16wKO #1-#3, surrounded by red circles) and their WT littermates (8wWT #1- #3 or 16wWT #1-#3, blue circles). The PCA plot showed that samples clustered based on their genetic backgrounds. Data represent n = 12. Component % variance; PC1 = 34.95%, PC2 = 19.48%. **B**) Heat map representation of differentially expressed genes in the mitral valves from either 8- or 16-wk *TAX1BP1*-KO mice or their WT littermates. 588 genes were differentially expressed in *TAX1BP1*-KO vs. WT littermates (P<0.03). Each column represents the expression profile of either the *TAX1BP1*-KO mice or WT littermates. Red and green colors indicate high and low expression levels, respectively, relative to the mean (see color bar). **C**) Volcano plot analysis of microarray revealed that 588 probes were significantly expressed more than 2-fold vs control. Red and green areas indicate significant increasing and decreasing changes in gene expression (p<0.03).

Principle component analysis, using two principle components, was conducted and the results were represented by a scatterplot (Fig. 1A). The data showed that the results for all samples from *TAX1BP1*-KO mice clearly deviated from those for control mice, indicating detectable differences in the gene transcription patterns of the two genetic backgrounds. A gene list was compiled on the basis of normalization and statistical analysis (P<0.03, logFC >1.0). Using these criteria, alterations in 588 gene expression profiles were identified. Unsupervised hierarchical clustering analysis (Cluster 3.0; Stanford University) of the 588 genes resulted in the separation of all *TAX1BP1*-KO from their paired WT controls. In total, 428 probes were upregulated and 160 were downregulated for a total of 24,000 genes (Fig. 1B). We then applied volcano plot analysis to identify the differences in mitral valve mRNA expression in *TAX1BP1*-KO mice and the controls. The plot showed a log2-fold change in mRNA expression between the two groups on the x-axis and the negative log of the t-test p-values on the y-axis. Each gene was represented by a single dot. Using the plot, we identified 588 probes that showed a more than 2-fold differential expression of mRNA when compared to the controls (p<0.03, Fig. 1C).

Tables 3 and 4 list the gene symbols, gene descriptions, fold changes, and p-value for all genes upregulated by more than 20-fold or downregulated by more than 5-fold. Most of the upregulated genes were primarily involved in inflammation. The gene showing the highest level of induction, *SAA3*, (i.e., 1,180 fold induction) along with *SAA1* (i.e., 61 fold, 10th induction) are well-known inflammatory markers in patients with autoimmune disease, chronic infection and cancer [25]. *SAA3* is also hyperinduced at the site of injury [26], inflammation [27] in mice experimental models. Additionally, genes related to immune modulation, including pathogen recognition, inflammation, chemotaxis [28]–[30], or tissue adhesion, degeneration and rearrangement [31], [32] were induced in the mitral valves of *TAX1BP1-*KO mice. The characteristics of the downregulated genes also suggested the link between inflammation and tissue degeneration (Table S1); for example, such as *WIF1*, a Wnt signaling suppressor; *UCMA*, a gene associated with cartilage development [33]–[35]. *EFCAB2* is a functional partner of the voltage-gated Ca^2+^ channel [36]. *TSC22D3* (also known as *GILZ*: a *G*lucocorticoid *I*nduced *L*eucine *Z*ipper) is an IL-10-inducible immune suppressor [37].

**Table 3:** Gene symbol, gene description, fold change and p-value for all genes up-regulated by >20-fold in *TAX1BP1*-KO mice.

SYMBOL	DESCRIPTION	Fold activation	adj.P. Val
SAA3	Serum amyloid A 3	1179.5	0.006
CHI3L1	Chitinase 3-like 1	361.0	0.006
HP	Haptoglobin	187.2	0.007
IL1B	Interleukin 1 beta	121.9	0.007
SPP1/OPN	Secreted phosphoprotein 1/Osteopontin	100.7	0.006
CCL2/MCP1	Chemokine (C-C motif) ligand 2/Monocyte chemotactic protein-1	81.7	0.021
CLEC7A/DECTIN1	C-type lectin domain family 7, member a/Dectin-1	81.0	0.005
SERPINA3G	Serine (or cysteine) peptidase inhibitor, clade A, member 3G	73.0	0.006
LCN2	Lipocalin 2	65.3	0.024
SAA1	Serum amyloid A 1	61.1	0.024
CXCL13/BLC	Chemokine (C-X-C motif) ligand 13/B lymphocyte chemo-attractant	52.9	0.0001
SLPI	Secretory leukocyte peptidase inhibitor	39.8	0.009
CLEC4D/DECTIN2	C-type lectin domain family 4, member d	39.6	0.006
TIMP1	Tissue inhibitor of metalloproteinase 1	37.4	0.024
CCL17/TARC	Chemokine (C-C motif) ligand 17/Thymus and activation regulated chemokine	35.2	0.020
CCL7	Chemokine (C-C motif) ligand 7	33.8	0.025
LGALS3/GALECTIN3	Lectin, galactose binding, soluble 3/Galectin-3	33.5	0.008
SIRPB1A	Signal-regulatory protein beta 1A	33.3	0.006
CHL1	Cell adhesion molecule with homology to L1CAM	32.4	0.027
CCL8	Chemokine (C-C motif) ligand 8	31.4	0.006
BCL2A1B	B-cell leukemia/lymphoma 2 related protein A1b	27.0	0.006
MEFV	Mediterranean fever	26.7	0.006
PLAC8	Placenta-specific 8	21.7	0.008
ZMYND15	Zinc finger, MYND-type containing 15	20.6	0.007
ITGAX	Integrin alpha X	20.0	0.006

Statistical significance (p<0.03) was calculated using the Empirical Bayesian method controlling for false discovery rate (FDR) <3% and logFC >1.0 on R statistical package (Version 2.15.1). Fold change represents a comparison between mean normalized signal intensity for control (n = 6) versus *TAX1BP1*-KO mice (n = 6).

**Table 4:** Gene symbol, gene description, fold change and p-value for all genes down-regulated by >5 fold in *TAX1BP1*-KO mice.

SYMBOL	DESCRIPTION	Fold suppression	adj.P. Val
TAX1BP1	Tax1 (human T-cell leukemia virus type I) binding protein 1	56.7	0.0000001
WIF1	Wnt inhibitory factor 1	11.1	0.0205
UCMA	Upper zone of growth plate and cartilage matrix associated	9.1	0.0173
EFCAB2	EF-hand calcium binding domain 2	8.0	0.0001
FAM107A/DRR1	Family with sequence similarity 107, member A/down-regulated in renal cell carcinoma 1	7.6	0.0219
TSC22D3	TSC22 domain family, member 3	7.3	0.0197
TAX1BP1	Tax1 (human T-cell leukemia virus type I) binding protein 1	7.2	0.0004
MAP3K6/ASK2	Mitogen-activated protein kinase kinase kinase 6	7.1	0.0212
6030422H21RIK	RIKEN cDNA 6030422H21 gene	6.8	0.0124
TSC22D3	TSC22 domain family, member 3	5.9	0.0240
PENK	Preproenkephalin	5.7	0.0119
CNTFR	Ciliary neurotrophic factor receptor	5.3	0.0104
COL11A2	Collagen, type XI, alpha 2	5.3	0.0069
RXFP3	Relaxin family peptide receptor 3	5.2	0.0197
NRXN1	Neurexin I	5.1	0.0110
CYTL1	Cytokine-like 1	5.0	0.0099

Statistical significance (p<0.03) was calculated using the Empirical Bayesian method controlling for false discovery rate (FDR) <3% and logFC >1.0 on R statistical package (Version 2.15.1). Fold change represents a comparison between mean normalized signal intensity for control (n = 6) versus *TAX1BP1*-KO mice (n = 6).

**Figure 2:**
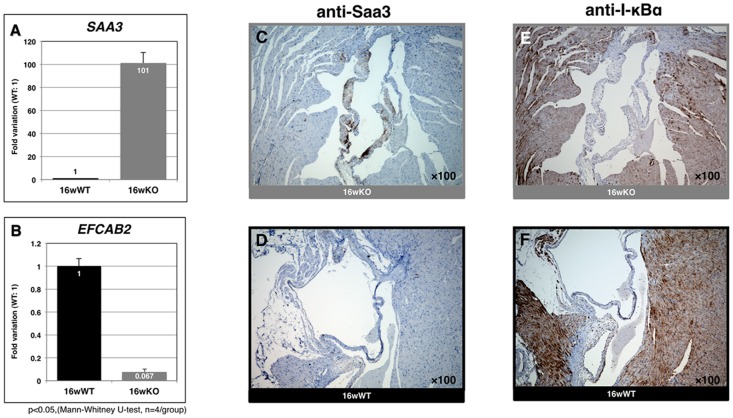
Validation of genes and proteins identified their expression alteration in the mitral valves of *TAX1BP1-*KO mice. RT-PCR validation of genes identified their expression alteration in the mitral valves of *TAX1BP1*-KO mice, **A**) *SAA3* **B**) *EFCAB2* respectively. Gray bar: *TAX1BP1*-KO, black bar: WT. Mitral valve specimens were prepared from 16-wk *TAX1BP1*-KO mice or their WT littermates and stained by anti-Saa3 antibody (**C** and **D**) or anti-I-κBα antibody respectively (**E** and **F**).

**Figure 3:**
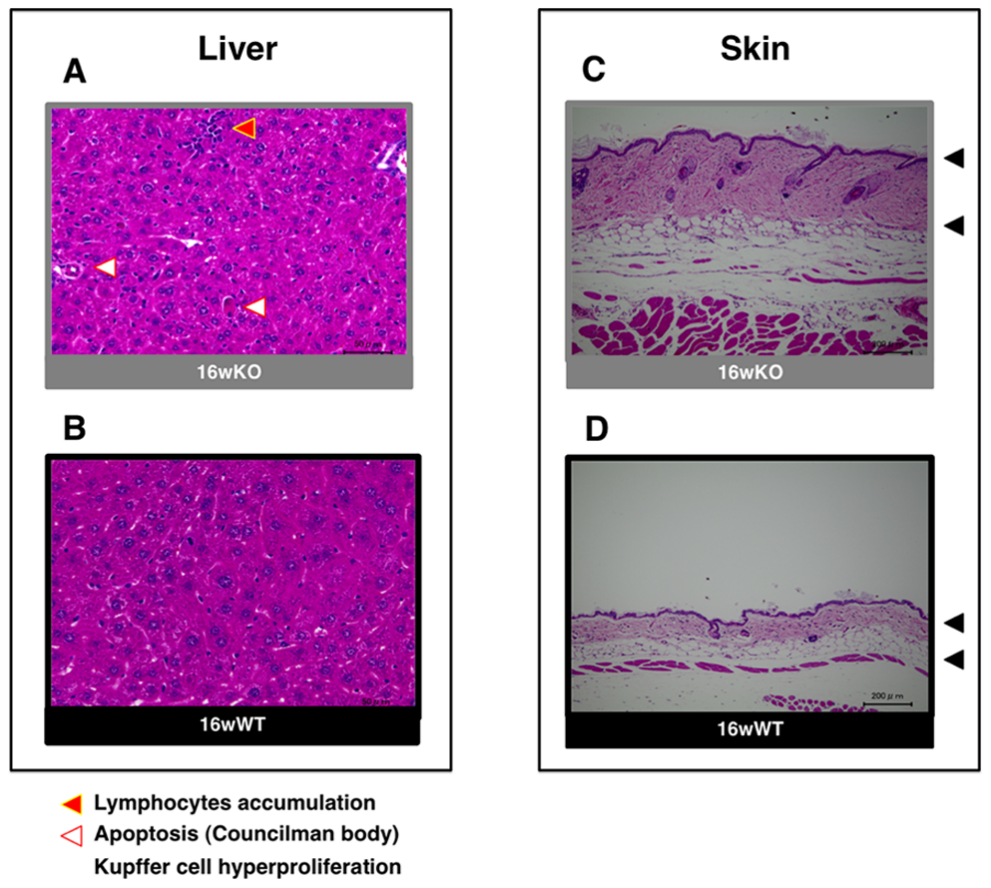
Inflammatory properties in the multiple organs of *TAX1BP1*-KO mice. The morphologic and functional alterations of the environments of liver (**A** and **B**) and skin (**C** and **D**) were also examined with HE-staining. Red and white triangles indicate accumulated lymphocytes and Councilman bodies respectively.

We further confirmed the microarray results for *SAA3* and *EFCAB2* by using RT-PCR (Fig. 2AB and Figure S1) and for Saa3 (induction) or I-κBα (reduction) by using immunostaining for mitral valve samples from 16-wk *TAX1BP1*-KO mice (Fig. 2C to F). In addition to these microenvironmental changes, broad-spectrum inflammatory effects, such as lymphocyte accumulation, apoptotic Councilman body formation, and Kupffer cell hyper proliferation in the hepatocyte (Fig. 3A), and thickening of the inflamed skin (Fig. 3C), were observed in 16-wk *TAX1BP1*-KO mice. Multiplex ELISA quantitation of the sera for homozygous or heterozygous *TAX1BP1*-KO and their WT littermates showed age-dependent development of systemic inflammation (Table S1). The levels of Il-6 and Cxcl1 were elevated more than 10- fold in the homozygous *TAX1BP1*-KO mice.

### Massive infiltration of inflammatory lymphocytes in the mitral valves of *TAX1BP1*-KO mice

**Figure 4:**
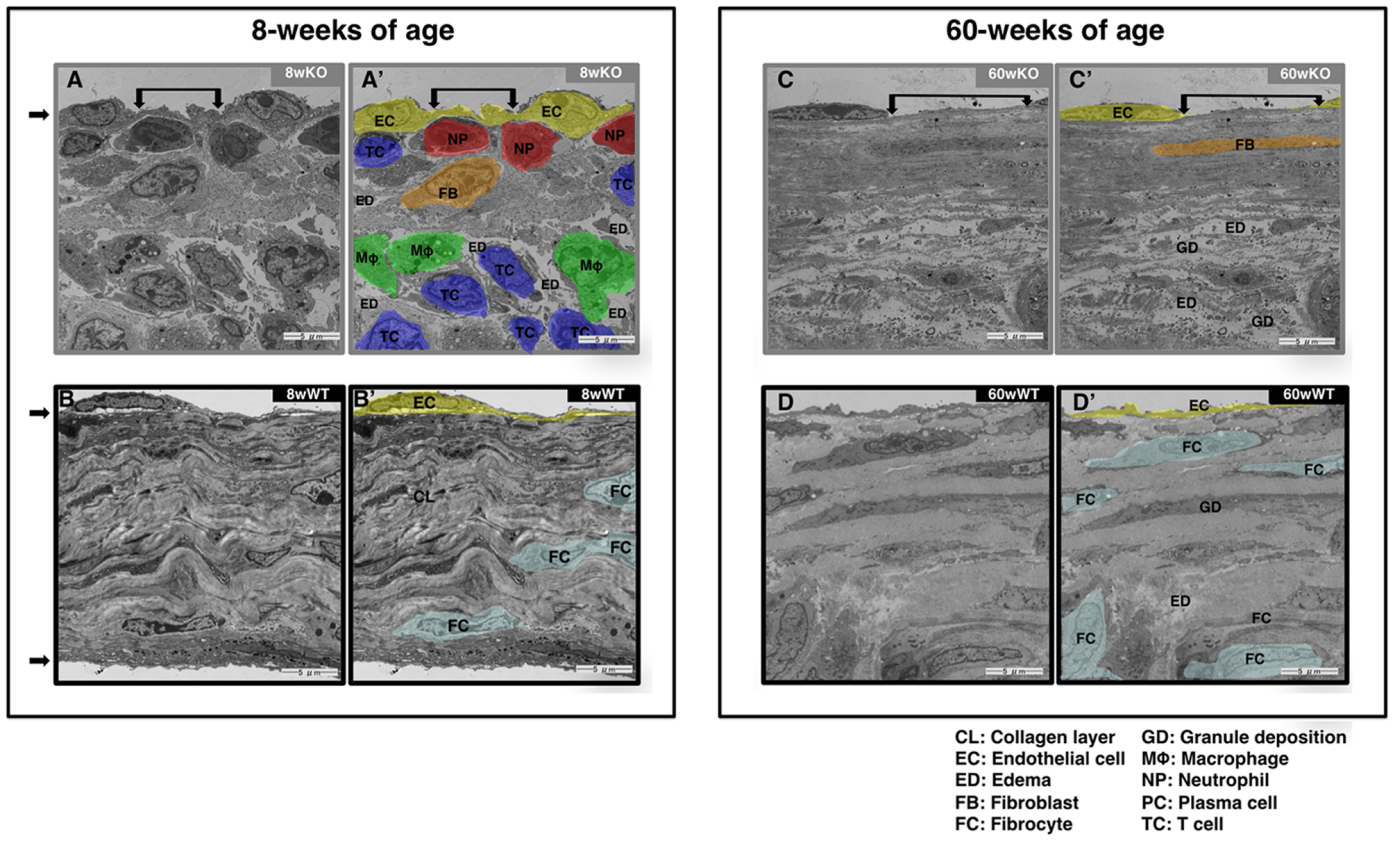
Massive infiltration of inflammatory cells causes severe tissue lesion in the mitral valves of *TAX1BP1*-KO mice. Electron microscopy examinations on the mitral valves of 8-, 16- and 60-wk *TAX1BP1*-KO mice (**A:** 8wKO, and **C:** 60wKO) and their WT littermates (**B:** 8wWT and **D:** 60wWT). See Figure S1 for details. Each panel was duplicated with colorized areas in specific cell types and abbreviated descriptions (Fig. 4A' to 4D'). Abbreviations, CL: Collagen layer; EC: Endothelial cell; ED: Edema; FB: Fibroblast; FC: Fibrocyte; GD: Granule deposition; MΦ: Macrophage; NP: Neutrophil; PC: Plasma cell; TC: T cell.

To obtain more detailed images of critical sites of inflammation, tissues obtained from the mitral valves of *TAX1BP1*-KO mice and their WT littermates at varying time points were examined with electron microscopy (Fig. 4). Surprisingly, the mitral valves *TAX1BP1*-KO mice showed extensive infiltration of lymphocytes, macrophages and neutrophils and tissue degeneration at only 8 weeks of age (Fig. 4A and 4A'), whereas the mitral valves of the WT littermates exhibited healthy collagen layers (Fig. 4B and 4B'). Extensive disruption of collagen layers and edema were observed at 60 weeks of age for *TAX1BP1*-KO mice (Fig. 4C, 4D and 4C', 4D').

### Enhanced inflammatory responses in *TAX1BP1*-KO mice after the LPS-stimulation

**Figure 5:**
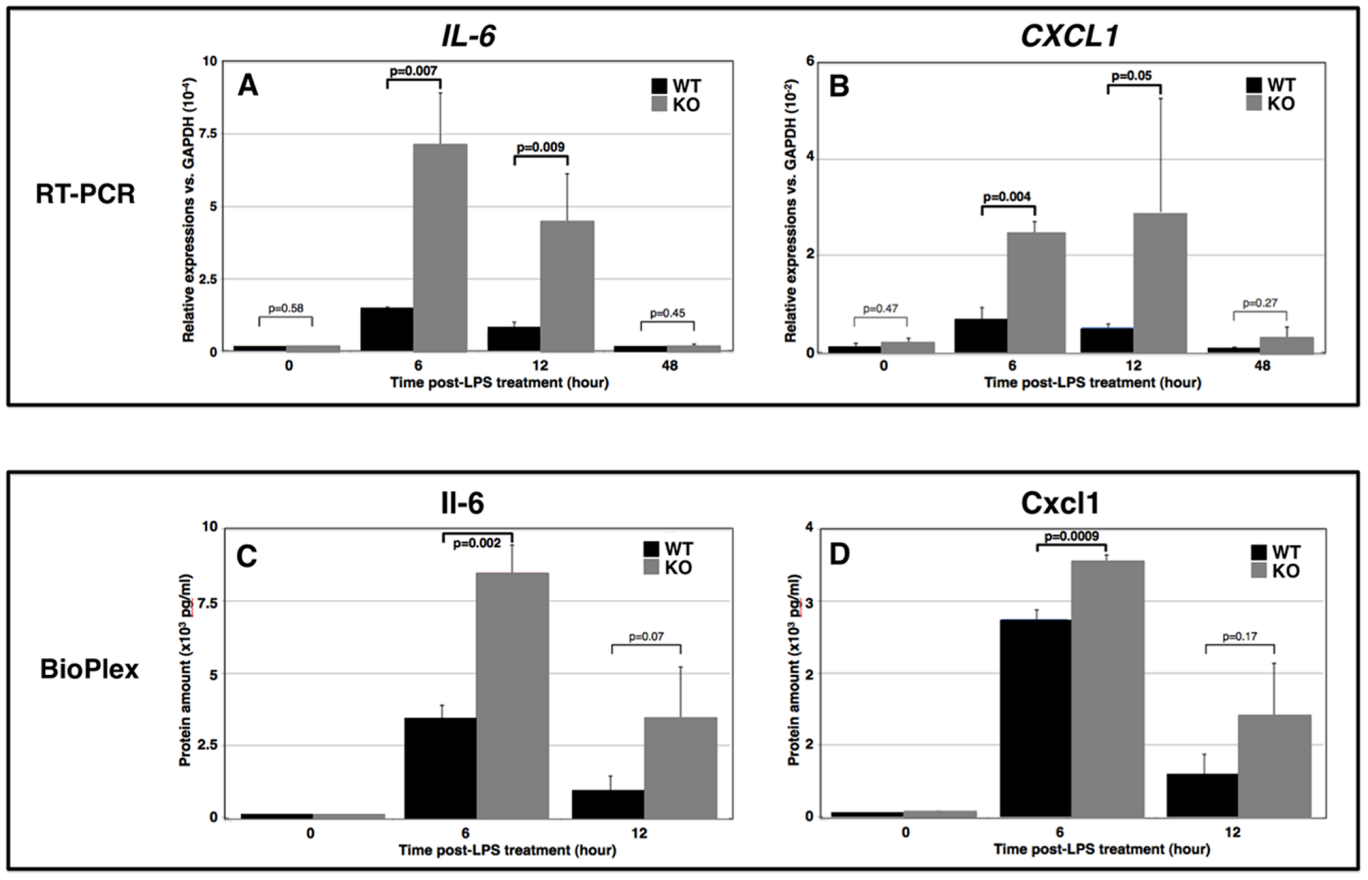
Enhanced expression of inflammatory genes after the LPS-stimulation to *TAX1BP1*-KO mice. 200 μg of *Salmonella typhimurium* lipopolysaccharide (LPS) in 100 μl sterile pyrogen-free saline were injected into a right footpads of *TAX1BP1*-KO or WT littermate mice. At the time 2, 6, 12, 24 and 48 hour post-injection (PT), each group of mouse were euthanized and tissues including serum, lymphocytes and eyes were collected. **A**) LPS-triggered induction of Tax1bp1 in eye tissue was monitored. Ten μg of cell lysates from WT BL6 mice at 2, 6, 12, 24 and 48 hour PT of LPS were probed with anti-Tax1bp1, -I-κBα and -Tubulin antibodies. **B, C**) Total RNAs of eye tissues from at 6, 12 and 48 hour PT of LPS to *TAX1BP1*-KO or WT littermates and their untreated controls were prepared and the expressions of *IL-6* and *CXCL1* were quantitated with RT-PCR. **D**, **E**) Sera from at 6, 12 and 48 hour PT of LPS to *TAX1BP1*-KO or WT littermates and their untreated controls were collected and the amount of Il-6 and Cxcl1 were quantitated with multiplex ELISA system (BioRad). Gray bar: *TAX1BP1*-KO, black bar: WT littermate.

In addition to the chronic inflammation, the acute-phase inflammatory response of *TAX1BP1*-KO mice was also examined. *Salmonella typhimurium* lipopolysaccharide (LPS) was injected into the footpads of *TAX1BP1*-KO mice and their WT littermates. Then, the mice were monitored, and the effects were recorded. We examined the kinetics of mRNA expression in those same eye tissues (Fig. 5A, B: tissue specific) and the translational products in the sera (Fig. 5C, D: systemic) of *IL-6* and *CXCL1* were monitored. Both data clearly indicate that a deficiency in *TAX1BP1* causes significantly enhanced inflammation in responses to LPS in *TAX1BP1*-KO mice.

### Amelioration of the inflammatory symptoms and the cardiac conduction defect of *TAX1BP1*-KO mice by antibiotic treatment and simultaneous *MyD88* deficiency

**Figure 6:**
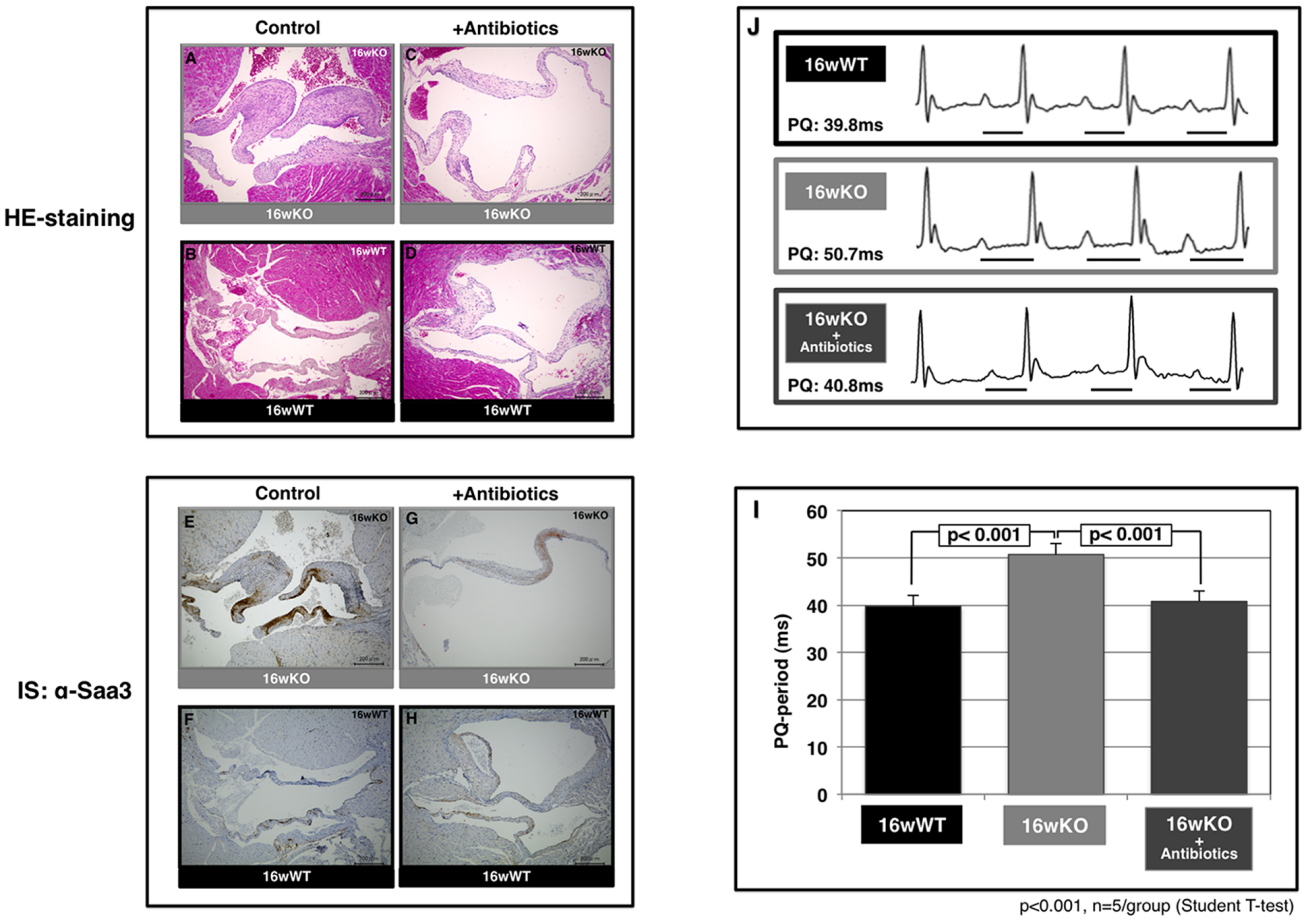
Amelioration of inflammatory valvulitis and conduction disturbance after the antibiotics treatment on *TAX1BP1-*KO mice. *TAX1BP1*-KO or WT littermate mice (male) were first raised with the normal diets and water for 4 weeks, and then, antibiotic treatment group (**C**, **D**, **G** and **H**, n = 5/group) provided ampicillin (1 g/L; Wako), vancomycin Hydrochloride (500 mg/L; Wako), neomycin trisulfate salt hydrate (1 g/L; Sigma-Aldrich), and metronidazole (1 g/L; Wako) in drinking water for 12 weeks based on a protocal of the commensal depletion (Rakoff-Nahoum S., Cell 2004). The non-antibiotics controls (**A**, **B**, **E** and **F**, n = 5/group) were equally raised and maintained except for antibiotics treatment. Each group of mice were anesthetized and sacrificed at the end of 16 weeks experimental period and histochemical representatives of each group were displayed with HE-staining (**A** to **D**) or anti-Saa3 immuno-staining (IS, **E** to **H**). **I**). Heart rhythms of 16-week-old *TAX1BP1*-KO treated with antibiotics over 12 weeks (male, n = 5/group) were monitored with telemetric electrocardiogram (12-lead ECG). **J**) The average values of PQ-intervals were compared with those of untreated *TAX1BP1*-KO mice and their WT littermates.

**Figure 7:**
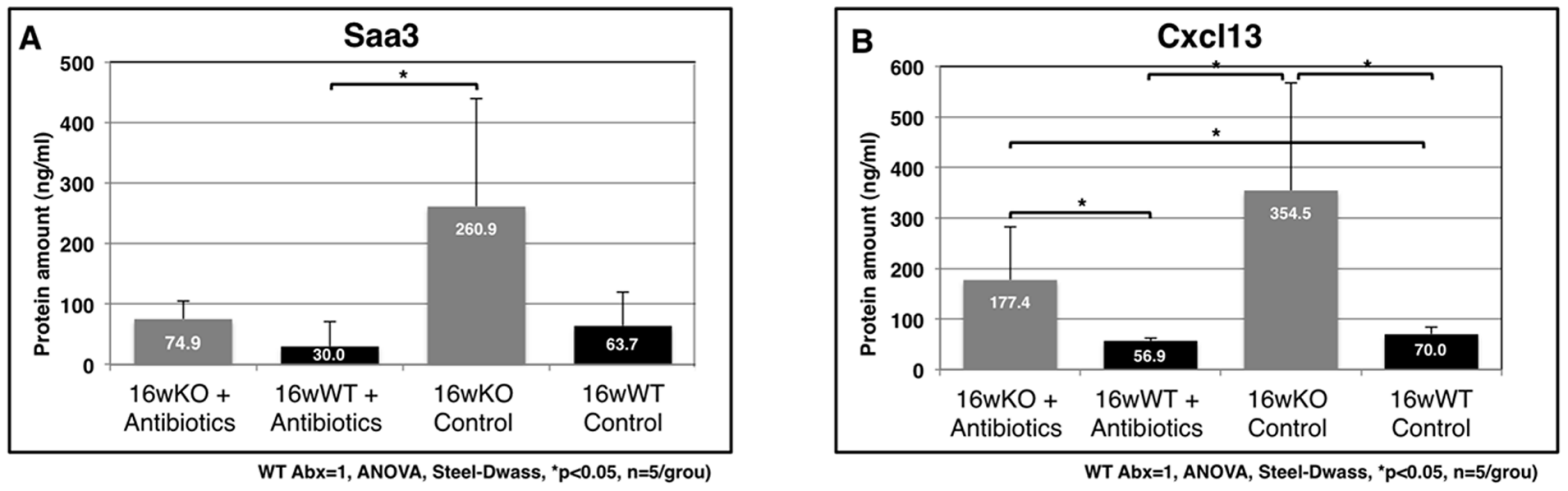
Reduction of the Saa3 and Cxcl1 expression in the sera of TAX1BP1-KO mice after the antibiotics treatment. ELISA quantitation of Saa3 (**A**) or Cxcl13 (**B**) of the sera on four groups were performed. Gray bar: *TAX1BP1*-KO mice, black bar: WT littermates (n = 5/group).

**Figure 8:**
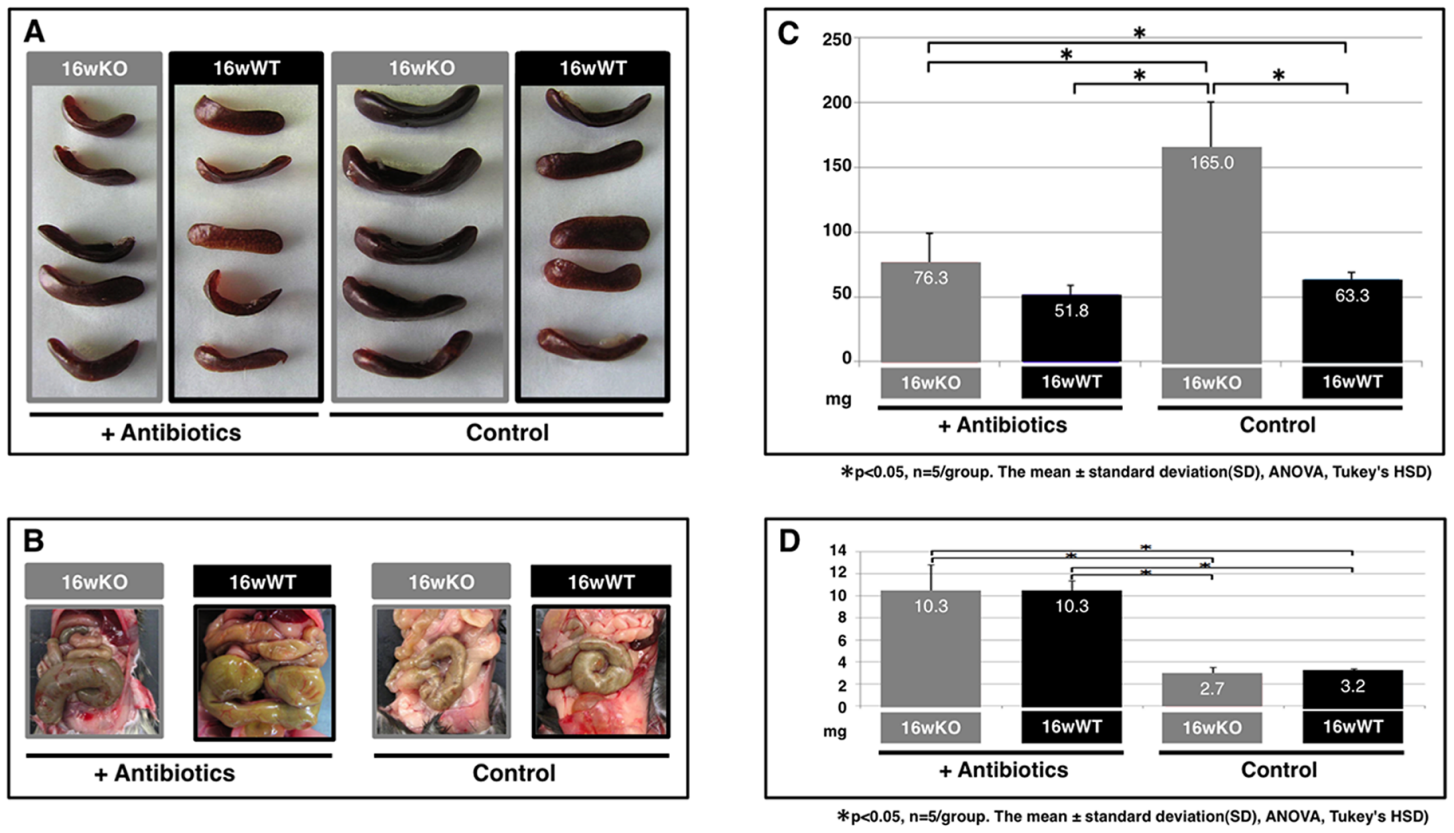
Splenic hypertrophy of *TAX1BP1*-KO mice and its cancellation by antibiotics treatment. Examinations on the spleen volume (**A**) and the area of cecum (**B**) were performed. The average values of spleen volumes (**C**) and cecum areas (**D**) were displayed (n = 5/group).

**Figure 9:**
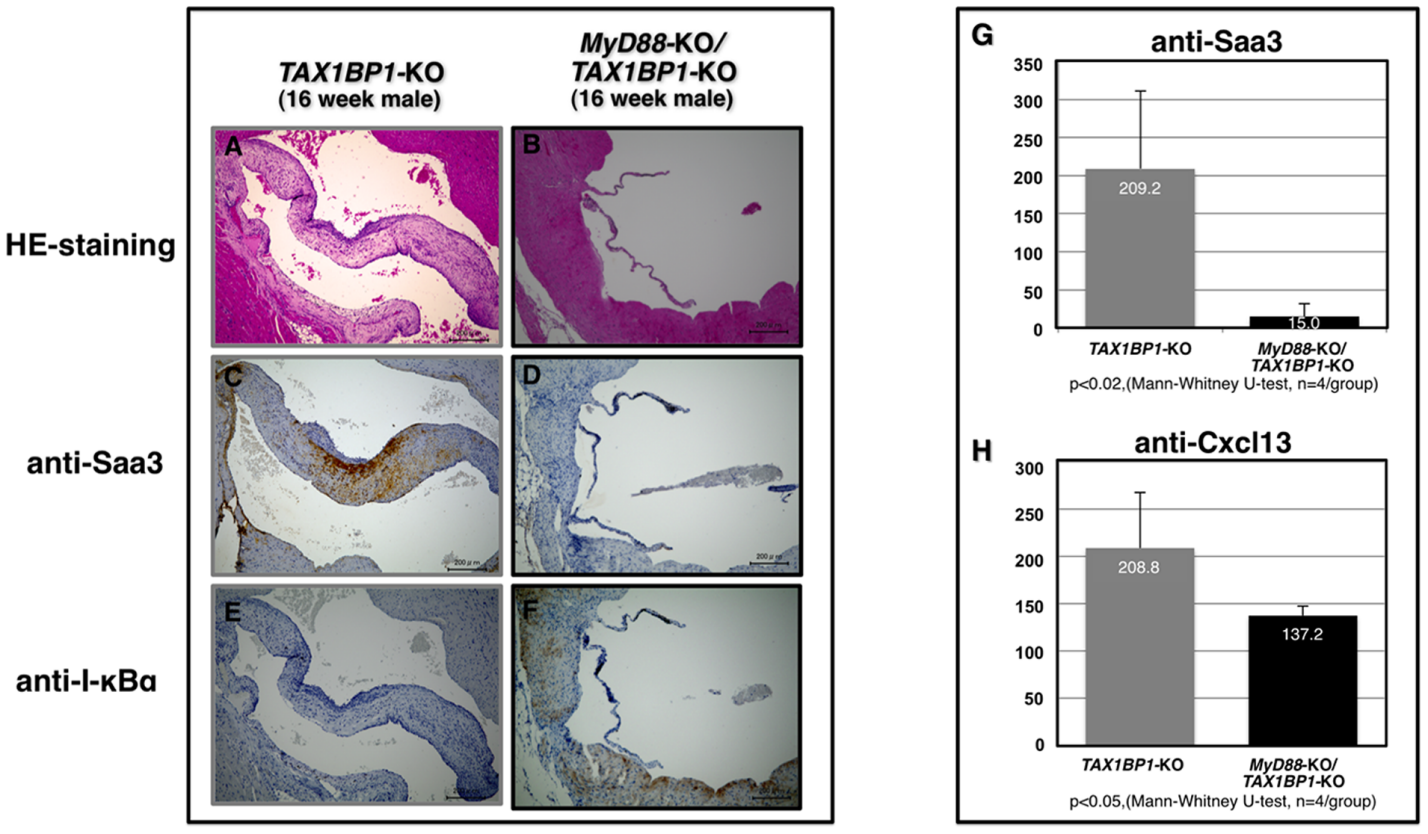
Cancelation of valvulitis in the *MyD88/TAX1BP1* double-KO mice. The HE-staining (A, B) and immunostaining of Saa3 (C, D) and I-κBα (E, F) were compared between *TAX1BP1*-KO and *MyD88/TAX1BP1-*KO mice. ELISA quantification of Saa3 (I) and Cxcl13 on the sera of both genetic background.

Microbial infections spontaneously cause severe endothelial inflammatory diseases such as rheumatic fever and Kawasaki disease [38]. At the subcellular level, modulation of the threshold of immune cell activation, differentiation, and immune cell activity in response to non-self or self antigens in *TAX1BP1*-KO mice (Fig. 1 and Tables 3 and 4) might evoke autoimmune profiles and heart dysfunction. To test this hypothesis, we examined the link between the commensal microbiota and mitral valvulitis and endocarditis in *TAX1BP1*-KO mice. When the mice were 4 weeks old, antibiotics were orally administered to all subjects over a 12-week period. The telemetric electrocardiogram profiles then sacrificed for the pathologic examination. Inflammatory hypertrophy (Fig. 6A) and extensive Saa3 staining (Fig. 6E) of the mitral valves in *TAX1BP1*-KO mice were abolished with antibiotic treatments (Fig. 6C and G); no changes were observed in their WT littermates (Fig. 6B, D, F and H). Extended PQ-intervals observed by telemetric electrocardiogram in *TAX1BP1*-KO mice (Fig. 6I, middle panel) were alleviated with the administration of antibiotics (Fig. 6I, bottom panel). The statistical significance of the differences in the PQ-intervals was tested (Fig. 6J). The antibiotic regimen also reduced the secretion of Saa3 and Cxcl13 in the sera of *TAX1BP1*-KO mice (Fig. 7A, B), and splenic hypertrophy of *TAX1BP1*-KO mice was almost nonexistent (Fig. 8A). Typical cecum thickening due to antibiotic treatment was also confirmed (Fig. 8B), and fecal microbes were completely disappeared under these conditions (data not shown). If the eradication of microbiota is the main reason for the amelioration of the symptoms in *TAX1BP1*-KO mice, we hypothesized that the disruption of the innate immune cascade could bring about similar results. We crossbred *TAX1BP1*-KO mice with *MyD88-*KO mice [20] and examined the morphological features or immunostaining profiles of marker proteins in the mitral valves of 16-week-old *TAX1BP1*-KO and *MyD88/TAX1BP1*-KO mice. *MyD88/TAX1BP1*-double knockout canceled hyperplasia (Fig. 9A, B), Saa3 induction (Fig. 9C, D) and I-κBα degradation (Fig. 9E, F). Comparisons of ELISA values for *TAX1BP1*-KO and *MyD88/TAX1BP1*-KO mice also indicated amelioration of the inflammatory response in *MyD88/TAX1BP1*-KO mice (Fig. 9G, H).

## Discussion

Chronic infection with a retrovirus can have a significant impact on the host immune system. In the case of HTLV-1 infection, the pathological features of the disease are influenced by multiple factors. While HIV causes immune deficiency in the host, HTLV-1 causes a wide range of inflammatory symptoms (HAM and HU) and, in some cases, immunosuppressive ATL, a malignant growth of regulatory T-lymphocytes [39], [40]. Furthermore, HAD patients frequently display impaired immune response such as an ineffective interferon response in HAM patients [41] and frequent development of dermatitis in ATL patients [42].

Multiple inflammatory symptoms, including cardiac valvulitis, dermatitis, and a hypersensitive response to endotoxins and inflammatory cytokines, were noted in our preclinical model involving *TAX1BP1-KO* mice. More importantly, *TAX1BP1-KO* mice died prematurely because of unknown mechanisms [8]. In this study, we discovered the hyper-induction of multiple inflammation-related genes including *SAA3*, *CHI3L1*, *HP*, *IL1B*, *SPP1/OPN*, and the significant reduction of *TSC22D3/GILZ* in the mitral valves and microenvironment deterioration in a progressive age-dependent manner for *TAX1BP1-*KO mice [43]–[47], the significant reduction of *EFCAB2* expression was highly implicated in functional defects of the heart [36].

HTLV-1-transgenic mice develop autoimmune symptom closely related to those observed for rheumatoid arthritis [48] or Sjögren's syndrome [49]. A rat model, infected with the HTLV-1 producing cell line, is known to develop HAM-like myelopathies in seronegative carrier rats [50]. A Tax1-transgenic mouse model, which specifically expresses Tax1 in T-lymphocytes, illustrates the development of aggressive ATL-like lymphoma with continuous invasion of lymphomatous cells into multiple organs such as the skin, liver and spleen [51], [52]. Subcutaneous inoculation of HTLV-1 transformed cells into NOG mice also results in ATL-like symptoms [53]. These transgenic/transplant models show symptoms similar to those found in human clinical cases. Furthermore, HTLV-1-driven inflammatory symptoms tend to occur in patients with HAD under normal host immune response conditions, while ATL-like symptoms develop under immunosuppressive conditions [54].

*TAX1BP1-*KO mice displayed invasive growth of lymphocytes into multiple organs (Fig. 3) and splenic hypertrophy (Fig. 8). We previously observed that transplantation of *TAX1BP1-*KO bone marrow to γ-irradiated normal mice resulted in the same inflammatory responses [8]. These results imply that *TAX1BP1-*KO model may be correlated with inflammatory HAD. The novelty of this system is identification of possible risk factors associated with vascular disease in HTLV-1 carriers [17], [18]. Preliminary electrocardiogram experiments using *TAX1BP1*-KO mice showed an abnormal prolongation of PQ intervals and/or atrioventricular conduction defects (Fig. 6I, J), which might cause fatal cardiac failure. Since the PQ interval and atrioventricular conduction highly depend on the functioning of voltage-dependent L-type Ca^2+^ channels, L-type Ca^2+^ channel function may deteriorate in the heart of *TAX1BP1*-KO mice. Of note, *EFCAB2*, a functional partner in the voltage-gated Ca^2+^ channel, was significantly downregulated in the cardiac tissue of *TAX1BP1*-KO mice (Table 4). Further studies are required to elucidate these defects caused in *TAX1BP1*-KO mice.

Intensive antibiotic treatment [24] for *TAX1BP1*-KO mice significantly ameliorated inflammatory symptoms (Fig. 6). *TAX1BP1*-KO mice crossbred with *MyD88-*KO mice showed similar results. Since the intrinsic role of Tax1bp1 is to inhibit unnecessarily activated innate immunity responses [8], a functional deficiency of Tax1bp1 through HTLV-1 infection can lead to similar symptoms in humans; that is, commensal microbiota can cause pseudo-Infective endocarditis symptoms [55]. The extent of the deficiency, however, is much more moderate than that of typical infective endocarditis (IE) [56].

A large population-based epidemiological study revealed that the prevalence of heart valve disease in the entire population of the United States is 2.5% [53]. IE is thought to result from the following sequence of events: (1) the formation of nonbacterial thrombotic endocarditis on the surface of a cardiac valve or elsewhere that endothelial damage occurs; (2) bacteremia; and (3) the adherence of the bacteria in the bloodstream to nonbacterial thrombotic endocarditis and proliferation of bacteria within a vegetation [57]. Viridans group streptococci are a part of normal skin, oral, respiratory, and gastrointestinal tract flora, and are responsible for ≥50% of community-acquired native valve IE cases [58]. Another review reported that 20% of IE cases originated from culture-negative or Enterococci [59]. Each of these epidemiological surveys clearly indicates the importance of prevention and control measures with regard to microbial infection and vegetation. However, it is still not known why IE is developed in limited population and it is not clear whether there are any differences in the frequencies of allelic polymorphisms in the immune response genes for IE patients?

In summary, HTLV-1 induces diverse forms of inflammatory disorders [60], [61], which may originate from the functional dysregulation of Tax1bp1. Single-nucleotide polymorphisms (SNPs) in *A20* or *RNF11*, catalytic partners of Tax1bp1, has have linked to many inflammatory diseases [19], [62], [63]. However, in the case of *TAX1BP1* SNPs, only one study has linked them to the head and neck cancer [64]. The genetic variations in *TAX1BP1* and its partners would provide novel insights on the pathogenic machinery of HADs.

## Supporting Information

Figure S1 **Validation of genes identified their expression alteration in the mitral valves of TAX1BP1-KO mice.** RT-PCR validation of genes identified their expression alteration in the mitral valves of *TAX1BP1*-KO mice, **A**) *CCL2* **B**) *CHI3L1* respectively. Gray bar: *TAX1BP1*-KO, black bar: WT. Mitral valve specimens were prepared as described in Fig. 2A. Primers and probes were as indicated.
(PDF)

Table S1 **Age-dependent induction of pro-inflammatory proteins in the sera of** ***TAX1BP1*** **-KO mice.** Sera from four different weeks of age (3, 8, 16 and 32) of *TAX1BP1* homozygous knockout (Homo-KO), heterozygous knockout (Hetero-KO) or their WT littermates were collected and examined with multiplex ELISA quantitation kit (Bio-Plex Pro™ Mouse Cytokine 23-plex Assay, BioRad). Each value is an average of four different samples.
(PDF)
