# Supplementary material for: Commensal Microbiota Contributes to Chronic Endocarditis in TAX1BP1 Deficient Mice
Source: PLoS One. 2013 Sep 27;8(9):e73205. doi: 10.1371/journal.pone.0073205 (PMC3785488; doi:10.1371/journal.pone.0073205)
Supplement: Figure S1 — Validation of genes identified their expression alteration in the mitral valves of TAX1BP1-KO mice. RT-PCR validation of genes identified their expression alteration in the mitral valves of TAX1BP1-KO mice, A) CCL2 B) CHI3L1 respectively. Gray bar: TAX1BP1-KO, black bar: WT. Mitral valve specimens were prepared as described in Fig. 2A. Primers and probes were as indicated. (PDF) [file pone.0073205.s001.pdf]

Suppl. 1. Validation of genes identified their expression alteration in the mitral valves of *TAX1BP1*-KO mice

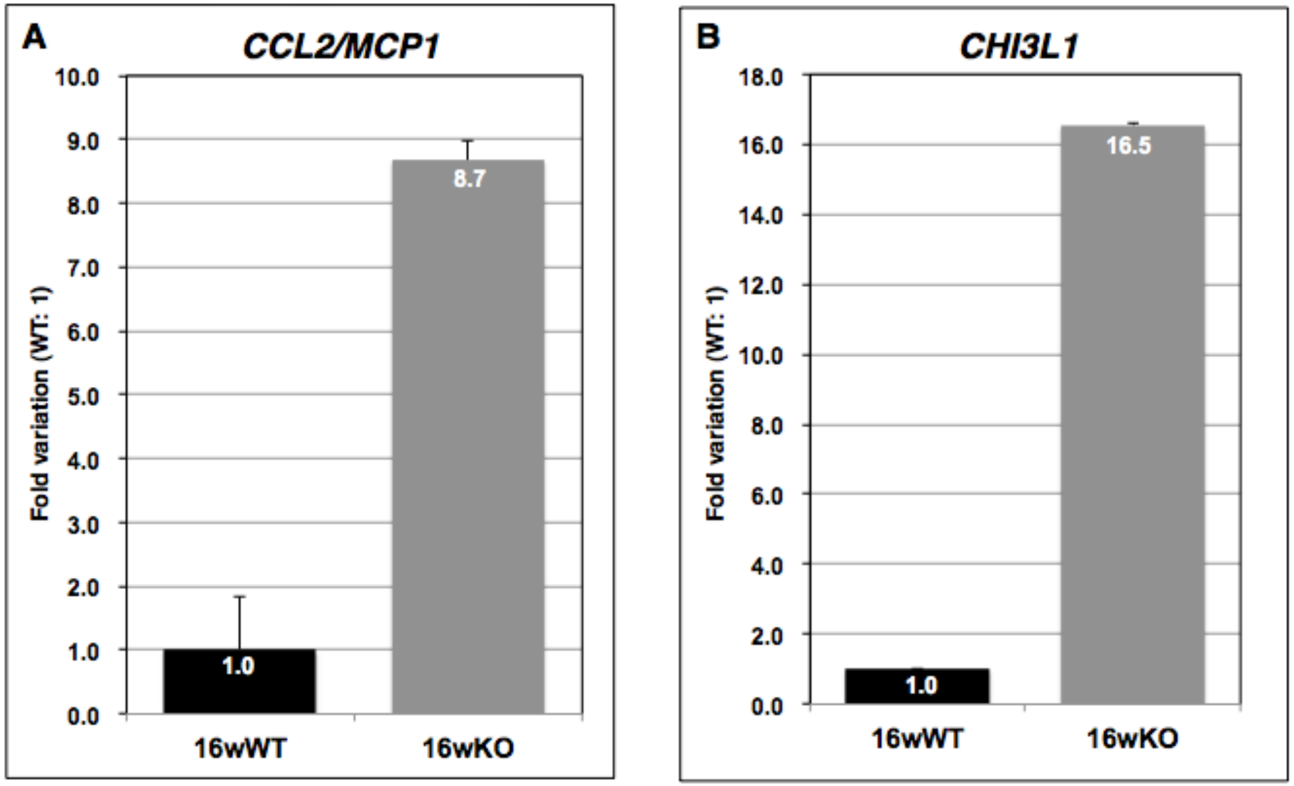

GENE	Primer 1	Primer 2	**RUP***
CCL2/MCP1	gatcatcttgctggtgaatgagt	catccacgtgttggctca	#62
CHI3L1	cgctgagcaggagtttctct	agcagtatttctccaccctgat	#20

RUP*: Roche Unversal Probe
