# Supplementary material for: Commensal Microbiota Contributes to Chronic Endocarditis in TAX1BP1 Deficient Mice
Source: PLoS One. 2013 Sep 27;8(9):e73205. doi: 10.1371/journal.pone.0073205 (PMC3785488; doi:10.1371/journal.pone.0073205)
Supplement: Table S1 — Age-dependent induction of pro-inflammatory proteins in the sera of TAX1BP1 -KO mice. Sera from four different weeks of age (3, 8, 16 and 32) of TAX1BP1 homozygous knockout (Homo-KO), heterozygous knockout (Hetero-KO) or their WT littermates were collected and examined with multiplex ELISA quantitation kit (Bio-Plex Pro™ Mouse Cytokine 23-plex Assay, BioRad). Each value is an average of four different samples. (PDF) [file pone.0073205.s002.pdf]

	3 weeks			8 weeks			16 weeks			32 weeks		
TAX1BP1 locus	Wild Type	Hetero-KO	Homo-KO	Wild Type	Hetero-KO	Homo-KO	Wild Type	Hetero-KO	Homo-KO	Wild Type	Hetero-KO	Homo-KO
IL-1a	1.0	0.7	0.6	1.0	1.3	0.8	1.0	**5.3**	**5.6**	1.0	**2.2**	**2.5**
IL-1b	1.0	1.0	1.1	1.0	1.3	0.7	1.0	1.5	**2.2**	1.0	**4.2**	**2.4**
IL-2	1.0	0.5	0.6	1.0	1.4	0.7	1.0	1.0	1.7	1.0	**4.5**	**2.3**
IL-3	1.0	0.3	0.3	1.0	0.6	0.9	1.0	1.2	1.9	1.0	1.6	1.0
IL-4	1.0	0.4	0.4	1.0	1.4	0.6	1.0	1.9	**2.1**	1.0	**4.6**	**2.4**
IL-5	1.0	0.9	1.1	1.0	1.3	1.1	1.0	**2.0**	**2.7**	1.0	**3.0**	1.4
IL-6	1.0	1.0	0.8	1.0	**4.6**	**8.7**	1.0	**2.6**	**8.0**	1.0	**7.6**	**51.3**
IL-9	1.0	1.2	1.3	1.0	1.3	1.1	1.0	1.2	1.4	1.0	1.7	1.5
IL-10	1.0	0.5	0.5	1.0	1.3	1.0	1.0	1.2	1.9	1.0	**4.8**	**2.7**
IL-12(p40)	1.0	0.8	0.7	1.0	1.6	**2.1**	1.0	**2.9**	**3.6**	1.0	**3.4**	**2.4**
IL-12(p70)	1.0	0.5	0.5	1.0	1.8	1.1	1.0	1.6	1.6	1.0	**3.0**	1.8
IL-13	1.0	0.6	0.7	1.0	1.2	0.9	1.0	1.1	1.8	1.0	**2.7**	**2.8**
IL-17	1.0	0.7	0.8	1.0	1.5	1.0	1.0	1.3	1.9	1.0	**2.8**	**2.7**
Eotaxin (CCL11)	1.0	0.8	0.9	1.0	1.4	1.0	1.0	0.8	1.3	1.0	**5.6**	**2.6**
G-CSF	1.0	0.3	0.3	1.0	1.1	1.1	1.0	**2.7**	**7.3**	1.0	1.4	**2.9**
GM-CSF	1.0	1.0	1.1	1.0	1.6	1.2	1.0	1.1	1.5	1.0	**5.1**	**2.6**
IFN-g	1.0	0.4	0.4	1.0	1.4	0.8	1.0	1.1	**2.1**	1.0	**4.5**	**2.0**
KC (CXCL1)	1.0	0.9	0.9	1.0	1.3	**3.6**	1.0	**2.4**	**13.7**	1.0	1.9	**5.6**
MCP-1 (CCL2)	1.0	0.6	0.7	1.0	1.4	1.2	1.0	1.2	**2.0**	1.0	**3.1**	**5.2**
MIP-1a (CCL3)	1.0	0.5	0.6	1.0	0.8	0.5	1.0	1.5	1.6	1.0	2.2	**3.0**
MIP-1b (CCL4)	1.0	0.3	0.3	1.0	1.6	0.8	1.0	1.2	**2.0**	1.0	**4.5**	**2.3**
RANTES (CCL5)	1.0	0.6	0.5	1.0	1.4	1.0	1.0	**2.0**	1.4	1.0	**3.4**	**2.3**
TNF-a	1.0	1.0	1.0	1.0	1.7	0.9	1.0	1.2	1.9	1.0	**3.5**	1.8
